# Association between Serum Uric Acid and Non-Alcoholic Fatty Liver Disease according to Different Menstrual Status Groups

**DOI:** 10.1155/2019/2763093

**Published:** 2019-11-23

**Authors:** Yanru Chen, Qiuping Huang, Ping Ai, Huamin Liu, Xueyu Chen, Xizhu Xu, Guoyong Ding, Yuejin Li, Xia Feng, Xiaohui Wang, Long Ji, Dong Li, Yong Zhou

**Affiliations:** ^1^School of Public Health, Shandong First Medical University & Shandong Academy of Medical Sciences, Taian, Shandong Province, China; ^2^Department of Emergency & Critical Care Medicine, Shanghai General Hospital, Shanghai Jiao Tong University, School of Medicine, Shanghai, China; ^3^Sanbo Brain Institute, Sanbo Brain Hospital, Capital Medical University, Beijing, China; ^4^Eye Hospital, Wenzhou Medical University, Wenzhou, Zhejiang, China

## Abstract

**Objective:**

The present study aimed to explore the association between SUA and NAFLD in women with different menstrual statuses.

**Methods:**

A total of 6043 women were selected from the Jidong and Kailuan communities for inclusion in the present study. The SUA levels of participants were divided into quartiles. NAFLD was determined by abdominal ultrasonography. Data from laboratory tests and clinical examination were collected, and basic information was obtained from standardized questionnaires. The menstrual status was stratified into menstrual period, menopause transition period, and postmenopause. Multivariate logistic regression models were used to determine the relationship between menstrual status, SUA, and NAFLD.

**Results:**

The levels of SUA in subjects with NAFLD in the menstrual period, menopause transition period, and postmenopause were 268.0 ± 71.1, 265.6 ± 67.8, and 286.7 ± 75.8 (mmol/L), respectively, and were higher than those in subjects without NAFLD. The adjusted odds ratios (ORs) with 95% confidence interval (CI) for NAFLD among participants in the menopause transition period and postmenopausal period were 1.10 (0.89–1.37) and 1.28 (1.04–1.58), respectively, compared with the menstrual period women. Compared to the lowest quartile of SUA, the adjusted ORs with 95% CI of the highest quartile for NAFLD were 2.24 (1.69–2.99) for females in the menstrual period, 1.92 (1.10–3.37) for females in the menopause transition period, and 1.47 (1.06–2.03) for females in postmenopause.

**Conclusions:**

Menstrual status was significantly correlated with NAFLD. High levels of SUA were associated with NAFLD in females during the three menstrual periods.

## 1. Introduction

Non-alcoholic fatty liver disease (NAFLD) is the leading cause of chronic liver disease, which has become an important public health issue [1]. Evidence has implied that the prevalence of NAFLD is higher in men than in women, but other studies indicated that it was higher in women than in men [2]. The prevalence of NAFLD was up to 30% in the general population and 26.3% in men [3–5]. In postmenopausal women, the prevalence of NAFLD was up to approximately 40%, which suggested a greater importance of NAFLD for postmenopausal women [6]. The development of NAFLD is also closely correlated with several chronic diseases, such as obesity, type 2 diabetes mellitus, dyslipidemia, and hypertension, which are components of metabolic syndrome (MetS) [7–9]. These involve the interaction among several signaling pathways. These include inflammation, oxidative stress, hepatocyte apoptosis, and insulin resistance associated with visceral adiposity and diabetes. [10–12]. Therefore, NAFLD is considered as a hepatic manifestation of metabolic syndrome. Over the past few years, studies have confirmed that SUA levels in patients with metabolic syndrome generally increase, and SUA levels increased with the number of metabolic syndrome-related disorders in patients [13].

Uric acid is the main product of purine metabolism with the catalysis of xanthine oxidoreductase [14]. SUA was associated with cardiovascular diseases (CVDs), of which one of the risk factors was NAFLD [15, 16]. Previous studies illustrated a positive association between SUA levels and the prevalence of MetS [7, 13, 17], but the relationship between NAFLD and SUA levels has been controversial in the literature, especially in women with different menstrual statuses [18–20]. Even more attractive was that hormone therapy can reduce SUA levels in postmenopausal females, indicating that high SUA levels were correlated with menopause [21]. Additionally, one study demonstrated that the uric acid level within normal range was associated with NAFLD in postmenopausal females, but not in premenopausal females, for which one possible reason is the decrease in hormone levels [22].

Although numerous studies have reported that NAFLD was correlated with elevated SUA in the general population and in males [5, 23, 24], few studies have been conducted on the correlation between NAFLD and SUA in women. Therefore, the present cross-sectional study was conducted to explore the association between SUA levels and NAFLD in females with different menstrual statuses.

## 2. Materials and Methods

### 2.1. Study Design and Participants

**Figure 1:**
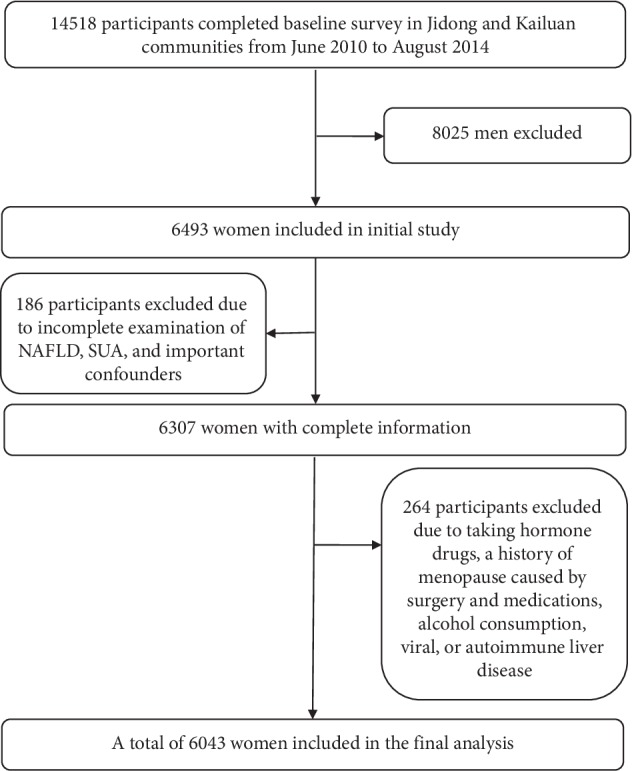
Flow chart of the study. NAFLD: non-alcohol fatty liver disease; SUA: blood uric acid.

The subjects in this cross-sectional study were employees and retirees recruited from the Jidong and Kailuan communities [25, 26] (Tangshan City, northern China) from 2010 to 2014. Among the 14518 participants, subjects with complete demographic and blood sample information were randomly selected to investigate the association between serum uric acid and non-alcoholic fatty liver disease according to different menstrual status groups. A total of 6493 subjects were included in the study after excluding 8025 men, 186 subjects with missing information on SUA, NAFLD, or important confounders, 264 women who met the following criteria were excluded: (1) history of treatment with exogenous estrogen or tamoxifen; menopausal history due to bilateral ovariectomy, drug use, or radiotherapy; (2) alcohol consumption more than 70 g/week for females; (3) other known history of chronic liver disease such as autoimmune hepatitis or viral hepatitis (HBsAg positive or anti-HCV antibody positive, etc.), and those using hepatotoxic drugs (Figure 1).

The study was conducted in keeping with the guiding principles of the Helsinki Declaration and was approved by the Ethics Committee of Jidong Oilfield Inc. Medical Centers and Kailuan General Hospital. Written informed consent was obtained from all participants.

### 2.2. Assessment of Potential Covariates

Standardized questionnaires, clinical examinations, and laboratory tests were used to collect basic information [27]. A standardized questionnaire to collect information on subjects' demographic characteristics was administered by well-trained interviewers. Demographic variables including age, gender, and education level; history of hypertension, diabetes mellitus, and dyslipidemia; and medications prescribed by physician were collected through questionnaire. According to self-reported information, alcohol use was defined as drinking at least 100 ml of alcohol (equivalent to 720 ml of beer or 240 ml of wine) per day for more than a year, and smoking status was classified as “nonsmokers or quitting more than one year” or “current smokers or quitting less than one year.” BMI were defined based on measured height and weight and calculated as weight (kg)/height (m^2^). Education was categorized into “illiteracy or primary,” “middle school,” or “university or above.” The average monthly income was divided into “≤¥3000, “¥3001–5000,” or “>¥5000.” The diagnosis of disease (hypertension, diabetes, and dyslipidemia) and measurement of body mass index, total cholesterol, triglyceride, high-density lipoprotein cholesterol, and low-density lipoprotein cholesterol have been described in our previous study [26, 28]. Hypertension was defined as the use of antihypertensive drugs, a self-reported history, diastolic blood pressure ≥90 mmHg, or systolic blood pressure ≥140 mmHg. Diabetes mellitus was defined as currently treated with insulin or oral hypoglycemic agent, presence of a history of diabetes, or fasting blood glucose level ≥7.0 mmol/L (126 mg/dL). Dyslipidemia was defined as current use of lipid-lower therapy, a self-reported history, or serum levels of TG ≥ 1.7 mmol/L, TC ≥ 5.18 mmol/L, HDL-C < 1.04 mmol/L, or LDL-C ≥ 3.37 mmol/L.

### 2.3. Determination of Serum Uric Acid

Blood samples were collected by venipuncture from the large antecubital veins in the morning after overnight fasting. All blood samples were stored in vacuum tubes containing EDTA (ethylene diamine tetraacetic acid), and SUA levels were determined using an autoanalyzer (Hitachi 747; Hitachi, Tokyo, Japan) with the uricase-peroxidase method in the laboratories at Jidong Oilfield Hospital and Kailuan General Hospital.

### 2.4. Diagnosis of NAFLD

According to the Chinese Association for the Study of Liver Disease and the Asia-Pacific Working Party on NAFLD [29] NAFLD was defined as the presence of at least two of the following findings (excluding excessive alcohol consumption and viral or autoimmune liver disease): diffusely increased echogenicity (‘bright') liver with liver echogenicity greater than kidney or spleen, vascular blurring, and deep attenuation of ultrasound signal. Abdominal ultrasonography was conducted by experienced radiologists who were not aware of the clinical characteristics and laboratory indicators using a high-resolution B-mode topographic ultrasound system with a 3.5 MHz probe (ACUSON X300, Siemens, Germany).

### 2.5. Assessment of Menstrual Status

Menstrual status was classified into menstrual period, menopause transition period, and postmenopause. The menstruation information was collected based on the participants' self-reports. For the classification of menstrual statuses, subjects were asked about the frequency and regularity of the menstrual cycle. Menopausal stages were defined as follows [30]: menstrual period: regular menstrual cycles within 7 days in the 22–35 day range; menopause transition period: changes in cycle length of ≥7 days for at least two consecutive menstrual cycles or amenorrhea for 3 to 11 months; and postmenopause: spontaneous menopause for more than one year.

### 2.6. Statistical Analysis

Continuous variables with normal distributions were expressed as the mean ± standard deviation (SD). Categorical variables were presented as frequencies and percentages. A *t*-test was used to test the difference with or without NAFLD for continuous variables, and the chi-square test was used for categorical variables in the different menstrual status groups. Nonparametric methods were used to compare ordinal variables and nonnormally distributed variables. Logistic regression analysis was used to examine the associations between menstrual status, SUA level, and NAFLD. The models were adjusted for the covariates of age, sex, education level, income, body mass index (BMI), hypertension, diabetes, hyperlipidemia, and waist circumference.

All statistical tests were 2-sided, with a significance level of *P* < 0.05. Statistical analyses were performed using SAS software, version 9.4 (SAS Institute Inc, Cary, North Carolina, USA).

## 3. Results

### 3.1. Characteristics of Participants

**Figure 2:**
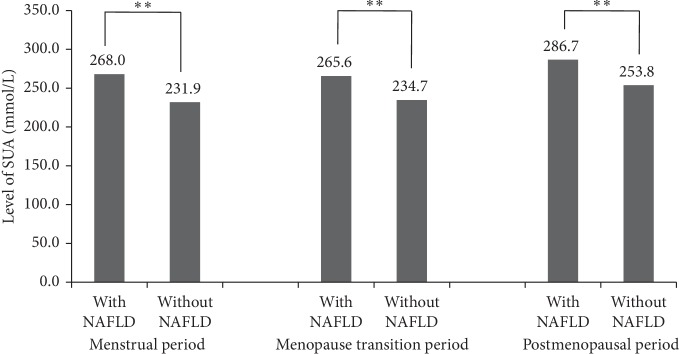
Level of SUA in participants with or without NAFLD stratified by menstrual status. NAFLD: non-alcohol fatty liver disease; SUA: blood uric acid. ^*∗∗*^*P* ≤ 0.05.

Table 1 shows the characteristics of participants with or without NAFLD in different menstrual status categories. The prevalence of NAFLD in the menstrual period, menopause transition period, and postmenopausal participants was 41.0% (824/3404), 12.1% (244/724), and 46.8% (941/1915), respectively. Participants with NAFLD were older and had different education levels. The income was significantly different between participants with or without NAFLD in the menstrual period group but not different in the menopause transition period and postmenopausal groups. Participants with NAFLD were more likely to have a history of hypertension and diabetes in different menstrual status groups (*P* < 0.05). Furthermore, they had higher levels of BMI, triglyceride (TG), total cholesterol (TC), low-density lipoprotein cholesterol (LDL-C), and waist circumference (WC) and lower levels of high-density lipoprotein cholesterol (HDL-C) (*P* < 0.001). The levels of SUA in subjects with NAFLD were higher than those without in different menstrual statuses (*P* < 0.05, Figure 2).

### 3.2. Association between SUA Levels and NAFLD in All Females

**Figure 3:**
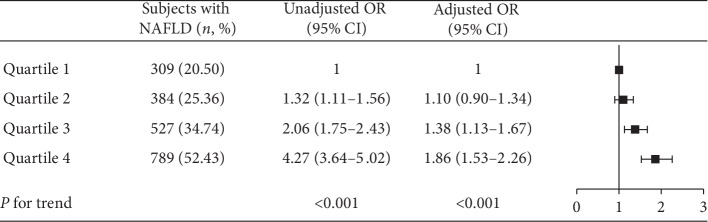
Association of SUA level with prevalence of NAFLD. Uric acid is quartile stratified. Adjusted OR: adjusted for age, education level, income, BMI, hypertension, diabetes, and hyperlipemia.

The prevalence of NAFLD was 20.50%, 25.36%, 34.74%, and 52.43% in the SUA quartiles, respectively. The ORs and 95% CI of NAFLD for subjects in the second, third, and fourth SUA quartiles were 1.32 (1.11–1.56), 2.06 (1.75–2.43), and 4.27 (3.64–5.02) in the unadjusted model, respectively, compared with those for subjects in the lowest quartile of SUA levels. The ORs and 95% CI of NAFLD in these quartiles were 1.10 (0.90–1.34), 1.38 (1.13–1.67), and 1.86 (1.53–2.26) in the adjusted model, respectively (Figure 3, *P* for trend across quartiles <0.001).

### 3.3. Association between Menstrual Status and NAFLD

**Figure 4:**
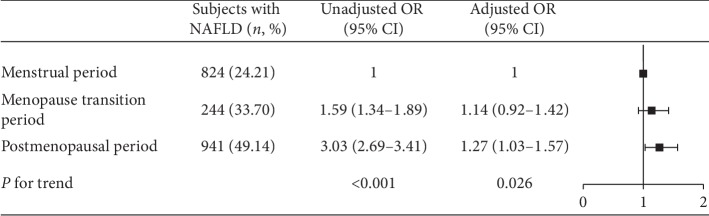
Association of menstrual status with prevalence of NAFLD. Adjusted OR: adjusted for age, education level, income, BMI, hypertension, diabetes, and hyperlipemia.

The prevalence of NAFLD was 24.21%, 33.70%, and 49.14% in the menstrual period, menopause transition period, and postmenopausal participants, respectively. The ORs and 95% CI for NAFLD in the menopause transition period and postmenopausal participants were 1.59 (1.34–1.89) and 3.03 (2.69–3.41) in the unadjusted model, respectively, compared to those for subjects in the menstrual period. The adjusted ORs and 95% CI of NAFLD in these menstrual statuses were 1.10 (0.89–1.37) and 1.28 (1.04–1.58), respectively (Figure 4, *P* for trend across menstrual status = 0.020).

### 3.4. Association between SUA Levels and NAFLD in Different Menstrual Status Groups

**Figure 5:**
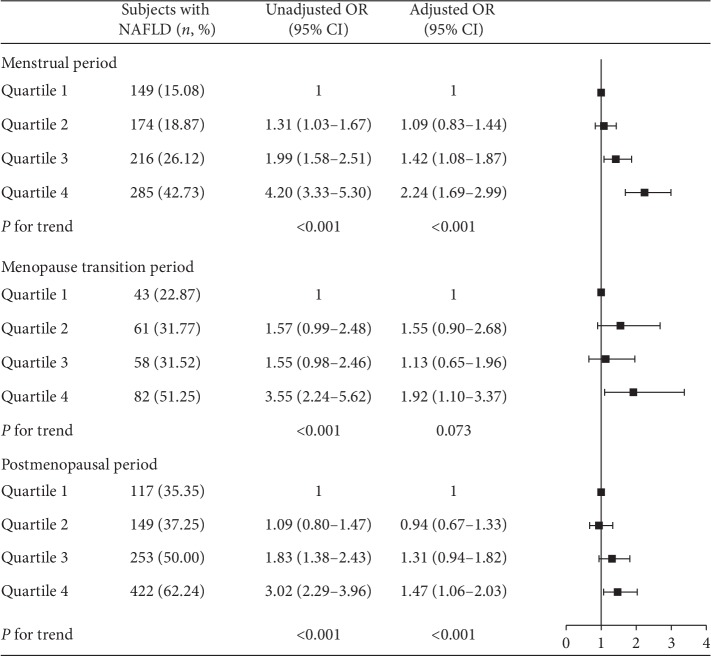
Association of SUA level with prevalence of NAFLD stratified by menstrual status. Uric acid is quartile stratified. Adjusted OR: adjusted for age, education level, income, BMI, hypertension, diabetes, and hyperlipemia. The interactive *P* value is 0.064.

As shown in Figure 5, the prevalence of NAFLD increased with SUA levels in women with different menstrual statuses. Participants in the fourth quartile of SUA had significantly increased ORs of 2.24 (95% CI: 1.69–2.99) for NAFLD in the menstrual period group, 1.92 (95% CI: 1.10–3.37) for NAFLD in the menopause transition period group, and 1.47 (95% CI: 1.06–2.03) for NAFLD in the postmenopausal group compared to the first quartile of SUA. A significantly increased trend of ORs for NAFLD was observed in the menstrual period and postmenopausal groups (Figure 5, *P* for trend across quartiles <0.001).

## 4. Discussion

The present study showed that SUA and menstrual status were associated with NAFLD in women. The prevalence of NAFLD progressively increased from the lowest quartile to the highest quartile of SUA. The risk of NAFLD increased approximately 40% and 80% in the third and fourth quartiles of SUA, respectively, after adjusting for potential confounding factors in all women. Furthermore, there were significantly increased risks for NAFLD in participants with high level of SUA even in different menstrual statuses.

Previous studies found that a high level of SUA was associated with NAFLD in males and in the general population [3, 4]. Li et al. found that elevated SUA was an independent risk factor for NAFLD [7]. The concentrations of SUA were demonstrated to be inversely associated with NAFLD remission in males [23]. The SUA was also implied to independently predict an increased risk for NAFLD in a prospective study [24]. In the present study, we observed a significantly increased risk of NAFLD in women with higher SUA levels, which was consistent with in the males.

The present study indicated that the risk of NAFLD was increased with SUA levels in women with different menstrual statuses. Studies have shown that hormones play a key role in this relationship and postmenopausal women with low levels of estrogen have a higher prevalence of NAFLD [31]. A previous study reported that women with NAFLD had significantly lower levels of serum estradiol than those without NAFLD and that the hormone profile was lower in postmenopausal women compared with premenopausal women [32]. One study showed that the relationship of SUA and NAFLD was significant in postmenopausal females, but not in premenopausal females [22]. However, there were significant relationships of SUA with NAFLD even in different menstrual statuses in our study. Estrogen deficiency may increase the risk of NAFLD. However, our study showed that the risk of NAFLD due to high uric acid in postmenopausal women with lower levels of estrogen was lower than that in premenopausal women with higher levels of estrogen, which was still statistically significant. This result suggested estrogen may be a potential mediator between uric acid and NAFLD. Further analyses of intermediary effects are needed to confirm this hypothesis.

The mechanisms including hyperleptinemia-induced oxidation stress and insulin resistance (IR) in patients with NAFLD can explain the positive correlation between SUA and NAFLD [33–35]. Leptin is the product of the obese (ob) gene and participates in the reabsorption of sodium and renal tubules, which may lead to elevated SUA levels [7]. Thus, elevated SUA levels may reflect a compensatory mechanism counteracting the increased oxidative stress associated with NAFLD. SUA can reduce endothelial nitric oxide (NO) bioavailability and supply to cells, and IR is then prompted accordingly [4]. Hyperuricemia resulting from IR was demonstrated to be associated with NAFLD. In addition, increased SUA and IR are characteristics of MetS, and NAFLD is the hepatic manifestation of MetS [34]. Thus, SUA is able to regulate lipid production and to foster the onset of metabolic disorders and NAFLD through multifaceted pathways. The past study has shown that the increase of estrogen level would lead to the change of renal tubule activity, impaired reabsorption, or increased secretion, so the uric acid clearance rate increased significantly [36]. In the postmenopausal period, the decrease of estrogen level would lead to the significant decrease of uric acid clearance rate [37]. In our study, we observed the highest prevalence of NAFLD in the quartile 4 of SUA in postmenopausal women, which may result from the decreased level of estrogen, thus increasing the uric acid level.

There are some limitations to our study. First, this is a cross-sectional observation study rather than an intervention study, so causality may not be inferred. Second, the diagnosis of NAFLD is mainly based on ultrasonography, which is not sensitive to the diagnosis of mild fatty degeneration. However, this method has been widely used in epidemiological studies of NAFLD because it is safe, noninvasive, and widely applicable and has good specificity and sensitivity in detecting hepatic steatosis. Third, the association between NAFLD and SUA was influenced by other unmeasured confounding factors. Finally, blood estrogen may be a potential mediator between uric acid and NAFLD, but we did not measure blood estrogen levels.

In conclusion, high SUA levels were associated with NAFLD among women, even in different menstrual statuses. Prevention of NAFLD is important for women in the lifetime. Also, uric acid can be a potential therapeutic target of NAFLD.

## Figures and Tables

**Table 1:** Characteristics of participants according to the status of NAFLD in different menstrual statuses.

Characteristics	Total	Menstrual period (*n* = 3404)		*P*	Menopause transition period (*n* = 724)		*P*	Postmenopausal period (*n* = 1915)		*P*
		With NAFLD	Without NAFLD		With NAFLD	Without NAFLD		With NAFLD	Without NAFLD	
*N*, (%)	6043	824 (41.0)	2580 (64.0)		244 (12.1)	480 (11.9)		941 (46.8)	974 (24.1)	
Age (years)	45.9 ± 12.8	41.5 ± 9.6	37.2 ± 8.9	<0.001	48.1 ± 6.9	44.8 ± 0.3	<0.001	60.1 ± 7.3	58.7 ± 8.5	<0.001
Education level, *n* (%)				<0.001			0.023			0.042
Illiteracy/primary	385 (6.4)	21 (2.6)	26 (1.0)		11 (4.5)	12 (2.5)		168 (17.9)	147 (15.1)	
Middle school	2991 (49.5)	381 (46.2)	857 (33.2)		147 (60.3)	252 (52.5)		668 (71.0)	686 (70.4)	
College or above	2667 (44.1)	422 (51.2)	1697 (65.8)		86 (35.3)	216 (45.0)		105 (11.2)	141 (14.5)	
Income, ¥/month, *n* (%)				<0.001			0.486			0.332
≤¥3000	3590 (59.4)	440 (53.4)	1164 (45.1)		157 (64.3)	292 (60.8)		750 (79.7)	787 (80.8)	
¥3001–5000	2130 (35.3)	336 (40.8)	1230 (47.7)		75 (30.7)	155 (32.3)		173 (18.4)	161 (16.5)	
>¥5000	323 (5.3)	48 (5.8)	186 (7.2)		12 (4.9)	33 (6.9)		18 (1.9)	26 (2.7)	
BMI (kg/m^2^)	23.9 ± 3.6	26.3 ± 3.6	22.3 ± 2.8	<0.001	26.8 ± 3.6	22.9 ± 2.7	<0.001	26.6 ± 3.3	23.4 ± 2.8	<0.001
Hypertension, *n* (%)	1632 (27.0)	264 (32.0)	229 (8.9)	<0.001	101 (41.4)	77 (16.0)	<0.001	567 (60.3)	394 (40.5)	<0.001
Diabetes, *n* (%)	418 (6.9)	82 (9.9)	28 (1.1)	<0.001	22 (9.0)	15 (3.1)	<0.001	180 (19.1)	91 (9.3)	<0.001
Hyperlipemia, *n* (%)	2001 (33.1)	369 (44.8)	363 (14.1)	<0.001	128 (52.46)	115 (24.0)	<0.001	609 (64.7)	417 (42.8)	<0.001
Total cholesterol (mmol/L)	4.6 ± 1.0	4.6 ± 0.9	4.2 ± 0.8	<0.001	4.7 ± 0.9	4.6 ± 1.0	0.106	5.3 ± 1.1	5.1 ± 0.9	0.004
Triglyceride (mmol/L)	1.4 ± 1.1	1.8 ± 1.2	1.1 ± 0.8	<0.001	1.8 ± 1.2	1.2 ± 0.9	<0.001	2.0 ± 1.5	1.4 ± 0.8	<0.001
HDL-C (mmol/L)	1.4 ± 0.4	1.3 ± 0.3	1.4 ± 0.4	<0.001	1.4 ± 0.4	1.5 ± 0.4	<0.001	1.4 ± 0.4	1.6 ± 0.4	<.0001
LDL-C (mmol/L)	2.5 ± 0.7	2.5 ± 0.6	2.2 ± 0.6	<0.001	2.6 ± 0.6	2.4 ± 0.7	0.002	2.9 ± 0.7	2.7 ± 0.7	<0.001
Waist circumference (cm)	82.4 ± 10.1	87.6 ± 9.6	77.8 ± 8.6	<0.001	89.1 ± 8.8	79.1 ± 8.5	<0.001	90.3 ± 8.9	82.5 ± 8.6	<0.001

NAFLD: non-alcohol fatty liver disease; SUA: blood uric acid; BMI: body mass index HDL-C: high-density lipoprotein cholesterol; LDL-C: low-density lipoprotein cholesterol.

## Data Availability

The data used to support the findings of this study are available from the corresponding author upon request.
